# Outcomes of autologous bone marrow mononuclear cells for cerebral palsy: an open label uncontrolled clinical trial

**DOI:** 10.1186/s12887-017-0859-z

**Published:** 2017-04-12

**Authors:** Liem Thanh Nguyen, Anh Tuan Nguyen, Chinh Duy Vu, Doan V. Ngo, Anh V. Bui

**Affiliations:** 1 Stem cells and Gene Technology Research Center, Vinmec International Hospital, 458 Minh Khai Street, Hanoi, Vietnam; 2 Department of Rehabilitation, Vinmec International Hospital, 458 Minh Khai Street, Hanoi, Vietnam; 3 Department of Diagnostic Imaging, Vinmec International Hospital, 458 Minh Khai Street, Hanoi, Vietnam

**Keywords:** Cerebral palsy, Stem cells

## Abstract

**Background:**

Stem cell therapy has emerged as a promising method for improving motor function of patients with cerebral palsy. The aim of this study is to assess the safety and effectiveness of autologous bone marrow mononuclear stem cell transplantation in patients with cerebral palsy related to oxygen deprivation.

**Methods:**

An open label uncontrolled clinical trial was carried out at Vinmec International Hospital. The intervention consisted of two administrations of stem cells, the first at baseline and the second 3 months later. Improvement was monitored at 3 months and 6 months after the first administration of stem cells, using the Gross Motor Function Measure (GMFM) and Modified Ashworth Score which measures muscle tone.

**Results:**

No severe complications were recorded during the study. After transplantation, 12 patients encountered fever without infections and 9 patients experienced vomiting which was easily managed with medications. Gross motor function was markedly improved 3 months or 6 months after stem cell transplantation than at baseline. The post-transplantation GMFM-88 total score, each of its domains and the GMFM-66 percentile were all significantly higher (*p*-value < 0.001). Muscle spasticity also reduced significantly after transplantation (*p*-value < 0.001). The therapy was equally effective regardless of sex, age and GMFCS level (*p*-value > 0.05).

**Conclusion:**

Autologous bone marrow mononuclear cell transplantation appears to be a safe and effective therapy for patients with cerebral palsy.

**Trial registration:**

ClinicalTrials.gov Identifier: NCT02569775. Retrospectively registered on October 15, 2015.

**Electronic supplementary material:**

The online version of this article (doi:10.1186/s12887-017-0859-z) contains supplementary material, which is available to authorized users.

## Background

Cerebral palsy is a non-progressive brain disorder affecting movement and posture. Its prevalence is 2.11 cases per 1000 live births (95% confidence interval [CI] 1.98–2.25) [1] . Management of cerebral palsy includes physical therapy, neurectomy, botulinum toxin A and medications, however these could not cure the disease [2]. Stem cell therapy has emerged as a promising method for improving motor function of patients with cerebral palsy.

Positive findings on motor improvement were demonstrated in animal models [3–5]. However, the mechanism of action by which stem cells exert their effects in cerebral palsy is still a topic of controversy. Some studies showed that stem cells could differentiate into neurons, oligodendrocytes and astrocytes [6–10]. Many other studies suggested that it was not the stem cell differentiation that would replace the injured cells but that the stem cells secreted trophic factors and cytokines, which modulate the micro environment, support anti-inflammation, cytoprotection and angiogenesis; generate myelin-producing cells and stimulate endogenous stem cells in the brain [11–15].

Stem cell therapy for cerebral palsy was shown to be safe and effective in human studies as well [11, 16–23]. Nevertheless, the number of studies on this promising intervention are still limited. More scientific evidence is needed to provide support for stem cell therapy as a standard of care for cerebral palsy.

The aim of this clinical study is to evaluate the safety and effectiveness of autologous bone marrow mononuclear cells (BMMNCs) in the management of cerebral palsy related to oxygen deprivation at Vinmec International Hospital, Hanoi, Vietnam.

## Methods

### Study design

An open label uncontrolled clinical trial of 40 patients aged 2 to 15 with cerebral palsy was conducted. The study commenced in April 2014 and completed in August 2015.

### Patient selection criteria

Patients diagnosed with cerebral palsy of any type related to oxygen deprivation at Vinmec International Hospital, Hanoi, Vietnam were included in this study. The exclusion criteria were GMFCS level I&II, epilepsy, hydrocephalus with ventricular drain, coagulation disorders, allergy to anesthetic agents, severe health conditions such as cancer, failure of heart, lung, liver or kidney and active infections.

### Intervention

The intervention included 2 intrathecal administrations of autologous Bone Marrow Mononuclear Cells (BMMNCs) at baseline and 3 months afterward conducted by certified anesthesiologists.

#### Isolation of BMMNCs

Bone marrow aspiration was performed under general anesthesia in the operating theatre. The volume collected depends on the patients’ body weight as followed: 8 ml/kg for patients under 10 kg; [80 ml + (body weight in kg - 10) × 7 ml] for patients above 10 kg but no more than 200 ml in total. BMMNCs were separated from the aspirate using the density gradient centrifugation using Ficoll [24]. The BMMNCs, Hematopoietic stem cells (CD34+ cells) were counted and checked for viability by Flowcytometry method.

#### Transplantation of BMMNCs

The BMMNCs were divided into two doses: one was given immediately after processing and the rest was stored in liquid nitrogen at minus 196 degree Celsius and administered 3 months after the first dose. The average numbers of mononuclear cells and CD34+ cells per 1 kg body weight transplanted for the first time were 27.2 × 10^6^ and 2.6 × 10^6^, respectively. The corresponding numbers for the second time were 17.1 × 10^6^ and 1.7 × 10^6^. The average of cell viability before the 1st and the 2nd injection was 97.8% % and 72%, respectively. The route of administration was intrathecal between the 4th and 5th lumbar vertebrae. Each dose of cells was mixed with saline to reach a volume of 10 ml for administration. An 18 gauge needle was used to ensure that cells were not sheared by the injection. The procedures were conducted in the recovery room and lasted for 30 min.

### Clinical assessment

Thorough clinical examinations were performed by a certified and experienced rehabilitation specialist at baseline, 3 months and 6 months afterwards with a special focus on the motor function. Children’s functional ability was classified based on Gross Motor Function Classification System (GMFCS) [25]. In order to evaluate the changes in motor function over time, a standardized observational tool namely the Gross Motor Function Measure (GMFM)-88 [26] was used. The GMFM-88 consists of 88 items categorized into five domains as followed: A. Lying and Rolling; B. Sitting; C. Crawling and Kneeling; D. Standing; E. Walking, Running and Jumping. The raw scores were entered into a computer program namely Gross Motor Ability Estimator to calculate the overall total scores, subtotal scores of each domains or to convert the GMFM-88 scores to GMFM-66 percentiles. The GMFM-66 percentile shows the relative motor function of a patient compared to children of the same age and GMFCS therefore it helped to exclude the interference of improvement with age [27]. Besides, muscle tone was assessed by Modified Ashworth Score [28].

GMFM-88 and GMFM-66 percentiles were primary outcomes. Modified Ashworth Score was secondary outcome.

### Laboratory and imaging diagnostics

Magnetic resonance imaging and electroencephalography of the brain, hematologic and biochemistry profile including HIV, HBV, and HCV tests were performed on all patients.

### Statistical analysis

Each individual is a unit of analysis. Paired t-test was used to compare the motor function and muscle tone at 3 months and 6 months with those at baseline. T-test or one-way ANOVA was used to further investigate changes in motor function and muscle tone according to patients’ characteristics. A *p*-value less than 0.05 was considered statistically significant. All statistical analyses were performed using STATA 11 (StataCorp, College Station, Texas).

## Results

### Patients’ characteristics

A cohort of 40 patients with cerebral palsy was included in this study. There were 33 males (82.5%) and 7 females (17.5%). The age median was 4 years old (range: 1–12 years old). The type of cerebral palsy observed in all patients was spastic (38 with bilateral paresis and 2 with unilateral paresis). The severity according to GMFCS was as followed: 2 (5%) with level III, 14 (35%) with level IV and 24 (60%) with level V. Three most common patterns found on MRI diagnostics are basal ganglia damage (39%), cortical/sub-cortical damage (8%) and periventricular white matter injury (8%). Patients’ characteristics are summarized in Table 1.

**Table 1:** Patients’ characteristics

Characteristics	*N* = 40 (100%)
Sex	
Male	33 (82.5%)
Female	7 (17.5%)
Age	
< 5 years old	25 (62%)
5–10 years old	13 (32%)
> 10 years old	2 (6%)
Type of Cerebral Palsy	
Bilateral spastic	38 (95%)
Unilateral spastic	2 (5%)
GMFCS	
Level III	2 (5%)
Level IV	14 (35%)
Level V	24 (60%)
MRI patterns	
Periventricular white matter injury	8 (22%)
Focal infarction	1 (3%)
Cortical/Sub-cortical damage	8 (22%)
Basal ganglia damage	14 (39%)
Miscellaneous	5 (14%)

### Adverse events

No complications were recorded during the procedure. After transplantation, mild fever was observed in 12 (30%) of the cases without any identified or suspected infections. Nine (22.5%) of the patients had intermittent vomiting which was also well managed with medications. Adverse events during and after stem cell transplantation are described in Table 2.

**Table 2:** Adverse events during and after stem cell transplantation

Adverse events	N (%)
Fever after transplantation	12 (30%)
Vomiting after transplantation	9 (22.5%)
Seizures after transplantation	0 (0%)
Other complications	0 (0%)

### Gross motor function and muscle tone before and after stem cell transplantation

Gross motor function improved remarkably after stem cell transplantation as compared to the baseline scores. The post-transplantation total GMFM-88 score and all of its domains were significantly higher (paired t-tests, *p*- value =0.001 in domain E and <0.0001 in the rest). Proportion of patients with improvement by GMFM domains were as follows: 100% in domain A (Lying and Rolling) and domain B (Sitting); 71% in domain C (Crawling and Kneeling); 64% in domain D (Standing); 38% in domain E (Walking, Running and Jumping). The GMFM-66 percentile measured only the relative motor function of the child as compared to other children of the same GMFCS and age. It was used to control for the possible improvement with age. The GMFM-66 percentile also improved significantly at 3 months after transplantation (Mean: 82.3%, 95% CI: [76.0; 88.5]) and 6 months after transplantation (Mean: 84.6%, 95% CI: [78.3; 90.8] as compared with baseline (Mean: 31.7%, 95% CI: [24.9; 38.5]). Muscle tone fell significantly from the mean modified Ashworth score of 3.4 at the baseline to 2.1 at 3 months and 2.0 at 6 months after the first transplantation (paired t test, *p*-value < 0.001). Gross Motor Function Measure and Muscle tone before and after stem cell transplantation are demonstrated in Table 3.

**Table 3:** Gross Motor Function Measure and Muscle tone before and after stem cell transplantation

	Baseline Mean [95% CI]	3 months post-transplantation Mean [95% CI]	6 months post-transplantation Mean [95% CI]
Total GMFM-88	16.7 [12.3; 21.1]	38.9 [32.2; 45.6]^*^	41.8 [34.8; 48.7]^*^
A. Lying & rolling	48.4 [41.2; 55.5]	87.8 [83.5; 92.1]^*^	91.7 [88.0; 95.3]^*^
B. Sitting	24.0 [16.5; 31.5]	55.1 [46.0; 64.3]^*^	59.5 [50.4; 68.7]^*^
C. Crawling & kneeling	8.7 [3.5; 13.8]	30.2 [18.9; 41.5]^*^	33.3 [21.4; 45.2]^*^
D. Standing	4.0 [0.9; 7.1]	17.9 [10.1; 25.7]^*^	20.2 [11.5; 28.8]^*^
E. Walking, running & jumping	1.2 [0.1; 2.4]	5.8 [2.2; 9.5]^**^	6.5 [2.5; 10.4]^**^
GMFM-66	24.8 [21.4; 28.1]	38.2 [34.6; 41.8]^*^	40.0 [36.3; 43.6]^*^
GMFM-66 percentiles	31.7 [24.9; 38.5]	82.3 [76.0; 88.5]^*^	84.6 [78.3; 90.8]^*^
Modified ashworth score	3.4 [3.2; 3.6]	2.1 [1.9; 2.3]^*^	2.0 [1.8; 2.2]^*^

*p-value < 0.0001 (paired t-test, compared to baseline)

**p-value = 0.001 (paired t-test, compared to baseline)

### Changes in gross motor function measure, muscle tone according to patients’ characteristics

Changes in gross motor function and muscle tone before and 6 months after stem cell transplantation were further analyzed by patients’ characteristics. The results are shown in Table 4.

**Table 4:** Changes in Gross Motor Function Measure, Muscle tone before and 6 months after stem cell transplantation according to patients’ characteristics

Characteristics	Change in GMFM-88 total score Mean [95% CI]	Change in GMFM-66 percentile Mean [95% CI]	Change in Ashworth score Mean [95% CI]
Sex	*p*-value = 0.09	*p*-value = 0.68	*p*-value = 0.88
Male	23.6 [19.3; 27.8]	52.2 [44.5; 60.0]	1.4 [1.2; 1.6]
Female	32.1 [22.6; 41.6]	56.0 [34.8; 77.2]	1.4 [0.9; 1.9]
Age	*p*-value = 0.07	*p*-value = 0.32	*p*-value = 0.89
< 5 years old	23.6 [19.4; 27.7]	52.5 [44.0; 61.1]	1.4 [1.1; 1.6]
5–10 years old	27.1 [19.4; 34.8]	52.4 [39.3; 65.5]	1.5 [1.1; 1.8]
> 10 years old	31.0 [−273.9; 336.0]	60.5 [−390.6; 551.6]	1.5 [−4.9; 7.9]
GMFCS	*p*-value = 0.23	*p*-value = 0.66	*p*-value = 0.90
Level III	33.5 [−49.1; 116.1]	63.5 [−44.5; 171.5]	1.5 [−4.9; 7.9]
Level IV	34.4 [27.4; 41.4]	57.6 [43.5; 71.7]	1.6 [1.3; 1.9]
Level V	18.9 [15.6; 22.2]	49.2 [40.5; 57.9]	1.3 [1.1; 1.5]

There was a trend for females to have better improvement in both gross motor function and muscle tone, but these findings were not statistically significant. Age and GMFCS level did not show any significant improvement of gross motor function and muscle tone.

## Discussion

In our study of the 40 patients with cerebral palsy related to oxygen deprivation, transplantation of autologous BMMNCs was apparently safe with no adverse events recorded during the procedure. During hospital stay after transplantation, 12 patients had mild fever but there were no signs of infections. Nine patients experienced intermittent vomiting, which was either self-limiting or relieved with medications. In a study of similar interventions on 40 patients by Sharma et al. demonstrated, 30% had vomiting and 2.5% had diarrhea [16]. In two other studies, there were no visible side effects or adverse reactions [11, 17].

GMFM improved remarkably after stem cell transplantation as compared to those in the baseline. The post-transplantation total GMFM-88 score, all of its domains and the GMFM-66 percentile were significantly higher. Muscle spasticity also reduced significantly after transplantation. Similar positive findings were also observed in other clinical studies [11, 16, 17]. In those studies, the improvement could be seen as early as 1 month and was most remarkable during the first 6 months after transplantation. In another study, positive changes continued till 18 months of follow-up but tended to slow down over time [11].

We further analyzed changes in gross motor function and muscle tone before and 6 months after stem cell transplantation by patient characteristics and doses of stem cells. The therapy was equally effective regardless of sex, age and GMFCS level.

Autologous stem cells were used in this study so there were no risks of rejection, anaphylaxis or side effects of immunosuppressive drugs that go along with allogenic transplantations. In a trial using allogenic stem cells from umbilical cord blood, severe adverse events such as pneumonia, influenza, urinary tract infection or even death were observed [29]. Different routes of cell administration have been used for patients with cerebral palsy such as intralthecal, intracranial and intravenous injection. In our study, stem cells were administered intrathecally so it was minimally invasive. Intracranial transplantation seems more targeted at the sites of lesions (i.e. the brain) but it was not chosen by our research team due to the invasive nature of this procedure. Moreover, in a study by Chen et al. using both methods of cell administrations, intra-cerebral surgery did not demonstrate a superior effect when compared to intrathecal transplantation [17]. Intravenous injection was not our route of choice. In animal models, it was observed that most of the IV transplanted cells were caught at the lung, spleen, kidney and intestine [30]. The strategy to transplant autologous BMMNCs intrathecally was also applied in other studies [11, 16, 20].

Our study has some limitations. It was an open label uncontrolled clinical trial as in some other studies [11, 16]. The follow-up time of 6 months was relatively short. There might be more long term improvement such as social-behavioral development or quality of life that to be monitored and evaluated.

## Conclusions

Based on the results of this study, we can conclude that autologous BMMNCs transplantation appears to be a safe and effective therapy for patients with cerebral palsy related to oxygen deprivation after 6 months of follow-up. Further studies are needed to examine the optimal regimen for this therapy and its long term effect ideally in double blind controlled trials.

## Additional file

Additional file 1: Stem cell transplantation for cerebral palsy. This is a dataset file of study on autologous bone marrow mononuclear cells for cerebral palsy. The data includes demographic data, gross motor function of patients with cerebral palsy such as GMFM-88 total score, GMFM-66 percentile, and complication figures. The dataset consists of 40 observations and 44 variables (XLSX 51 kb)

## Abbreviations

BMMNCs: Bone marrow mononuclear cells
GMFCS: Gross motor function classification system
GMFM: Gross motor function measure
MSCs: Mesenchymal stem cells
